# Supplementary material for: Diffractive Refractometer Based on Scalar Theory
Source: Polymers (Basel). 2023 Mar 23;15(7):1605. doi: 10.3390/polym15071605 (PMC10096849; doi:10.3390/polym15071605)
Supplement: Supplementary file 1 [file polymers-15-01605-s001.zip › polymers-2269814-supplementary.pdf]

Supporting Information for

Diffractive refractometer based on scalar theory

Marcella Salvatore[†,‡], *Francesco Reda*[‡], *Fabio Borbone*[§], *Pasqualino Maddalena*[‡,†] *and Stefano L. Oscurato*[‡,†,*]

[†]Centro Servizi Metrologici e tecnologici Avanzati (CeSMA), University of Naples "Federico II", Complesso Universitario di Monte Sant'Angelo, Via Cintia 21, 80126, Naples, Italy.

[‡]Physics Department "E. Pancini", University of Naples "Federico II", Complesso Universitario di Monte Sant'Angelo, via Cinthia 21, 80126, Naples, Italy.

[§]Department of Chemical Sciences, University of Naples "Federico II", Complesso Universitario di Monte Sant'Angelo, Via Cintia, 80126 Naples, Italy

***Corresponding author**

Email: stefanoluigi.oscurato@unina.it

Parametric dependence of diffraction efficiency and sensitivity for surface relief sinusoidal diffraction gratings in scalar diffraction theory

Model for refractive index dispersion in transparent materials

Sellmeier equation successfully describe the refractive index dependence on light wavelength for transparent materials exhibiting normal dispersion.[1,2] This model is a simplified version of the Lorentz model of oscillating dipoles for the dielectric function of materials, where dissipation terms are neglected (no absorption). The general form of the Sellmeier dispersion equation is: $n^2(\lambda) - 1 = A\lambda^2/(\lambda^2 - \lambda_0^2)$,where A and λ_0 are the amplitude and the resonance wavelength of the oscillating dipoles, respectively. The validity of this model is restricted to wavelength regions far from the resonance wavelength (located typically in the UV).

To evaluate the parametric dependence of the diffraction efficiency of a sinusoidal grating on the grating amplitude h and light wavelength λ within scalar diffraction theory, we consider PMMA and TiO2 as exemplary low and high dispersion transparent materials in the VIS-NIR wavelength range.

According to Ref.[3] (PMMA) and Ref. [4] (TiO_2), Sellmeier models for the two materials in this range, shown in Figure S1, are:

$$n(\lambda)_{PMMA}{}^2 - 1 = \frac{1.1819\lambda^2}{\lambda^2 - 0.11313} \qquad \textit{eq. (S1)}$$

$$n(\lambda)_{TiO2}{}^2 - 5.913 = \frac{0.2441\lambda^2}{\lambda^2 - 0.00803} \qquad \textit{eq. (S2)}$$

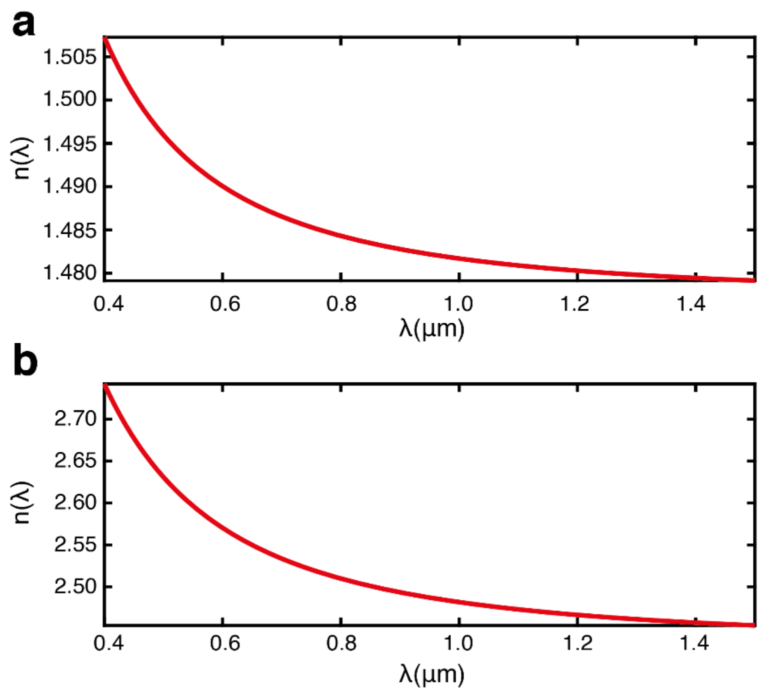

Figure S1. Sellmeier model for the refractive index dispersion n=n(λ) of PMMA(a) and TiO2(b) in region λ=0.4-1.5 μm.

Dependence of diffraction efficiency on wavelength and grating amplitude

According to scalar diffraction theory, the diffraction efficiency in the mth order produced by a sinusoidal surface relief grating, in the ideal case depicted in Figure 1a on the main text, is described in terms of Bessel functions of first kind and order m as:

$$\eta_m(\lambda, h, n) = \left| J_m \left[\pi(n-1)\frac{h}{\lambda} \right] \right|^2 \qquad \textit{eq. (S3)}$$

By using a model for the refractive index dispersion $n=n(\lambda)$, the diffraction efficiency η_m in this model parametrically depends only on the wavelength and the grating amplitude $\eta_m = \eta_m(\lambda,h)$. This consideration allows a deeper analysis of the combined influence of the grating structural parameter h and light wavelength on the diffraction behavior of the grating. Figure S2a and Figure S2a show this relation in the fist five diffraction orders ($m=0$, ± 1, ± 2) sinusoidal gratings made of PMMA and TiO_2, respectively in parametric range ($h = 0 \div 1.0$ μm; $\lambda = 0.4 - 1.0$ μm) compatible with the experimental conditions of the experiment in the main text. Sellmeier relations in eq. (S1) and eq. (S2) have been used in the simulation of the dispersion for the two materials.

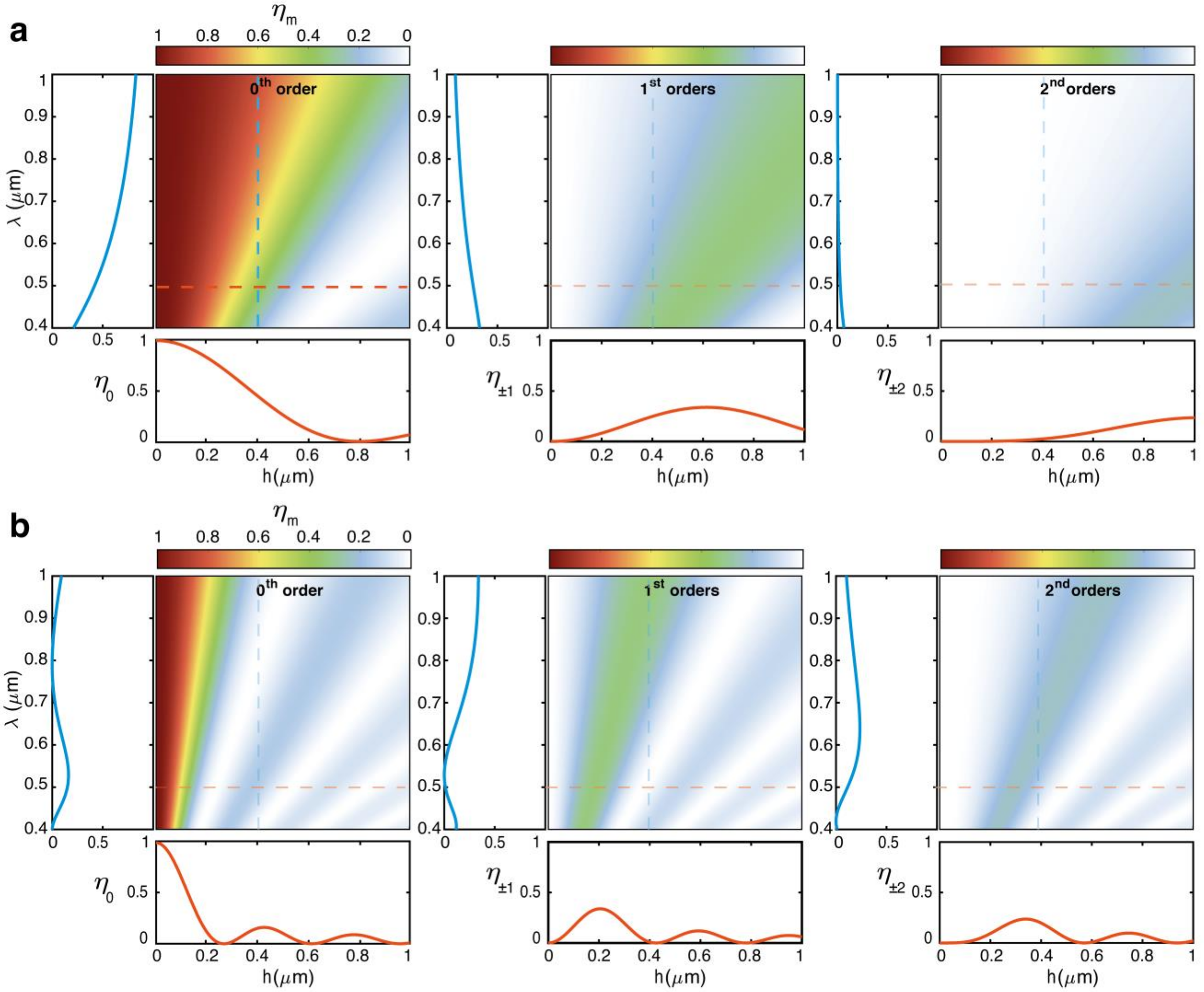

Figure S2. Parametric dependence of the diffraction efficiency in the first five orders produced by a sinusoidal surface relief grating in scalar diffraction theory on the light wavelength λ and grating amplitude h. Sellmeier models for the refractive index dispersion of PMMA (a) and TiO2 (b) have been used. For deeper insight, each panel shows also single parameter dependence for fixed wavelength (λ=0.5 μm, red curves) and fixed grating amplitude (h=0.4 μm, blue curves)

Dependence of efficiency sensitivity on wavelength and grating amplitude

The derivatives of eq. (S3) with respect to the parameters λ and h can be used to define *sensitivity* functions for the variations of the diffraction efficiency with the wavelength and grating amplitude:

$$S_{m,h}(\lambda, h) = \left|\frac{\partial \eta_m(\lambda, h)}{\partial h}\right| \qquad \textit{eq. (S4)}$$

$$S_{m,\lambda}(\lambda, h) = \left|\frac{\partial \eta_m(\lambda, h)}{\partial \lambda}\right| \qquad \textit{eq. (S5)}$$

Figure S3 and Figure S4 show the plot of the grating amplitude sensitivity (panels a) and the wavelength sensitivity (panels b) for PMMA and TiO_2 gratings, respectively. While the amplitude grating sensitivity can be used to design the best grating amplitude optimizing the sensitivity to diffraction efficiency variations in the desired wavelength range for the diffractive refractometer, the wavelength sensitivity gives more details on the expected effectiveness of the method when a single grating with fixed amplitude is used to investigate a large wavelength range, as in the case of the experiment in Figure 4 of the main text. It is worth noting that higher dispersive materials (TiO_2 here) show less sensitivity of diffraction efficiency variations with the wavelength, when compared to lower dispersive materials (e.g. PMMA).

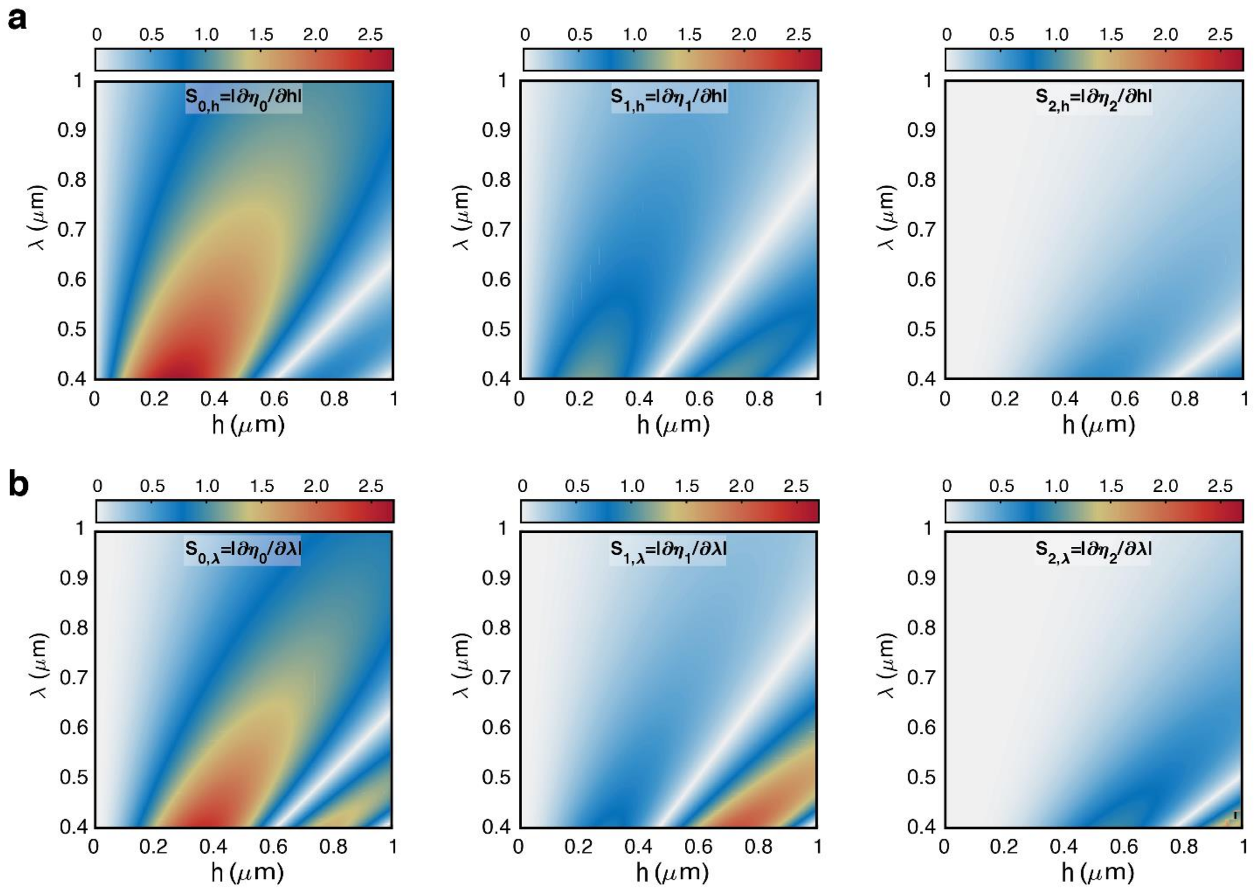

Figure S3. Sensitivity for PMMA in the first five diffraction orders of the scalar diffraction theory for a sinusoidal surface relief grating. (a) Sensitivity $S_{m,h}$ to the parameter h and (b) sensitivity $S_{m,\lambda}$ to the parameter λ. Sellmeier model (eq. (S1)) for refractive index dispersion has been used for n(λ) in these calculations.

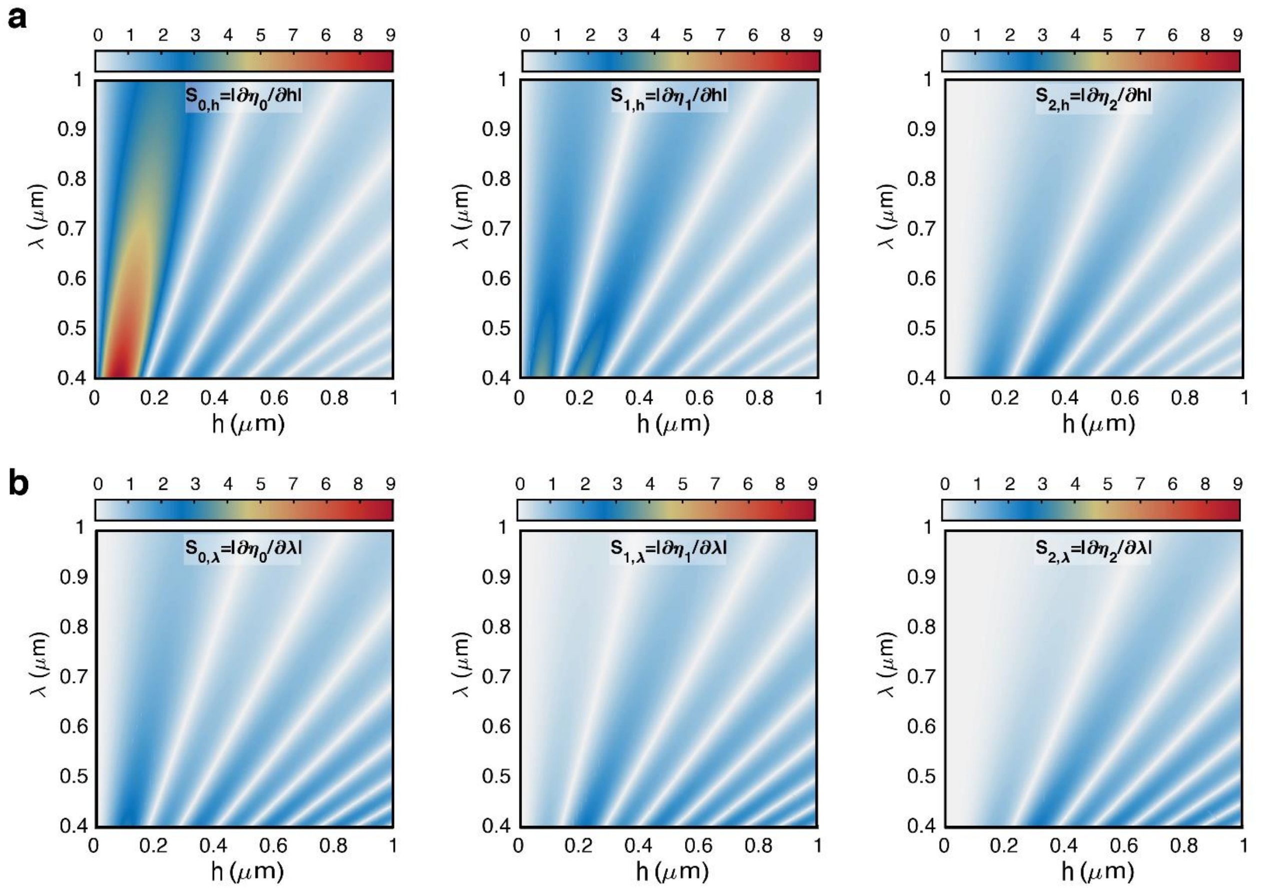

Figure S4. Sensitivity for TiO_2 in the first five diffraction orders of the scalar diffraction theory for a sinusoidal surface relief grating. (a) Sensitivity $S_{m,h}$ to the parameter h and (b) sensitivity $S_{m,\lambda}$ to the parameter λ. Sellmeier model (eq. (S2)) for refractive index dispersion has been used for n(λ) in these calculations. The higher refractive index and dispersion of TiO_2 with respect to PMMA results in several narrow bands of high sensitivity in the considered parameter space.

References

(1) *Spectroscopic Ellipsometry for Photovoltaics: Volume 1: Fundamental Principles and Solar Cell Characterization*; Fujiwara, H., Collins, R. W., Eds.; Springer Series in Optical Sciences; Springer International Publishing: Cham, 2018; Vol. 212. https://doi.org/10.1007/978-3-319-75377-5.

(2) Harland G. Tompkins; Eugene A. Irene. *Handbook of Ellipsometry*; William Andrew Publishing: Norwich, 2005.

(3) Sultanova, N.; Kasarova, S.; Nikolov, I. Dispersion Properties of Optical Polymers. *ACTA PHYSICA POLONICA A* **2009**, *116*, 585–587. https://doi.org/10.12693/APhysPolA.116.585.

(4) DeVore, J. R. Refractive Indices of Rutile and Sphalerite. *J. Opt. Soc. Am., JOSA* **1951**, *41* (6), 416–419. https://doi.org/10.1364/JOSA.41.000416.
